# Knowledge towards cervical cancer screening and associated factors among urban health extension workers at Addis Ababa, Ethiopia: facility based cross-sectional survey

**DOI:** 10.1186/s12885-021-07952-z

**Published:** 2021-03-05

**Authors:** Tiruneh Ararsa, Niguse Tadele, Yohannes Ayalew, Debela Gela

**Affiliations:** 1 Oncology Nurse, Black Lion Specialized Hospital, College of Health Science, Addis Ababa University, P.O. Box: 5657, Addis Ababa, Ethiopia; 2 School of Nursing & Midwifery, College of Health Science, Addis Ababa University, P.O. Box: 100686, Addis Ababa, Ethiopia; 3 School of Nursing & Midwifery, College of Health Science, Addis Ababa University, P.O. Box: 4412, Addis Ababa, Ethiopia

**Keywords:** Knowledge, Cervical cancer, Health extension workers, Screening, Perception, Ethiopia

## Abstract

**Background:**

Cervical cancer is preventable and remains a leading cause of avoidable death among women in the world. In a developing country, the knowledge of screening for cervical cancer behavior still very low. However, little is known about the knowledge towards cervical cancer screening of urban health extension workers in Ethiopia. This study aimed to assess knowledge towards cervical cancer screening and associated factors among urban health extension workers in Addis Ababa, Ethiopia, 2020.

**Methods:**

In this cross-sectional study, 312 urban health extension workers completed the survey in the Amharic language. Data collected using a structured questionnaire in a face-to-face interview. Descriptive and logistic regression analyses were conducted using SPSS version 26.

**Results:**

The mean age of the urban health extension workers was 20.41 ± 3.73 years and 55.1% were married. The majority of the participants (75.6%) had diploma educational level, and 38.1% of them had 1–2 years of work experience. More than half (51.6%) of the participants had poor knowledge about cervical cancer screening. Participants with work experience of 5–6 years (AOR = 4.32: 95% CI = 1.71,10.94) and those who had a monthly income of 5000–10,000 ETB (AOR = 3.75: 95% CI = 1.49,9.41) and greater than > 10,000 ETB (AOR = 3.08: 95% CI =1.06, 8.98) were positively associated with knowledge towards cervical cancer screening among urban health extension workers, *p*-value< 0.05.

**Conclusion:**

This study indicated that the knowledge towards cervical cancer screening of urban health extension workers was inadequate. Urban health extension workers’ work experiences and monthly income were found to be independent predictors of the knowledge towards cervical cancer screening of respondents. Therefore, urban health extension workers with low work experiences and those with small monthly income could be targeted for cervical cancer screening information and training interventions.

## Introduction

Cervical cancer is a potentially preventable and treatable form of cancer so that morbidity and mortality could be reduced with early detection and effective management [1]. However, it remains to be a leading cause of morbidity and avoidable death among women worldwide with more than 5 hundred thousand new cases each year, and about 80% of the cases arise in low-income countries [2–4]. In Ethiopia, cervical cancer ranks as the second common cause of cancer death next to breast cancer among women [5, 6] with an incidence and mortality rates of 26.4 and 18.4 / 100,000 [7].

In developed countries, the incidence of cervical cancer, the incidence of cervical cancer has decreased from 2.8 to 2.3 per 100,000 women [8] due to effective screening programs [9]. However, in developing countries like Ethiopia [10], shortage of screening facilities, financial issues, cultural factors, and lack of awareness limit the uptake of cervical cancer screening [11].

In Ethiopia, the coverage of cervical cancer screening remains very low, ranging from 2.0–20.2% in the urban areas and 0.4–14.0% in rural areas [2, 12, 13]. Lack of knowledge/ awareness [13–16], low-risk perception [13, 15], limited access, lack of financial resources, the symptomless nature of cervical cancer and the stigma associated with the disease [16], negative perception towards cervical cancer screening [17–20] were the most commonly mentioned barriers towards cervical cancer screening.

According to the World Health Organization (WHO) recommendation, for women who are living in developing countries; cervical cancer screening should begin at the age of 21 and continued to preforming screening once every 1–5 years and alone testing with cytology every 3 years of women aged 30 to 65 years [20, 21]. Ethiopia adopted cervical cancer prevention and control guideline from the WHO and recommend women to get screening for cervical cancer at least every 5 years and currently, both arranged and opportunistic cervical cancer screening are available [22, 23].

Besides, recent studies have shown that over 80% of cervical cancer cases are detected at advanced stages of cancer due to a low level of knowledge about the disease as well as lack of awareness of available screening methods [6, 24] and indicated that knowledge of women on cervical cancer, cervical cancer screening and perceived susceptibility might influence a women’s decision and uptake of cervical cancer screening [21, 25, 26]. Hence, promoting awareness about the disease and prevention activities amongst communities is crucial to saving the lives of women from cervical cancer.

The Urban Health Extension Program was started in 2009 in Ethiopia to create health equity by generating demand for essential health services through the provision of health information at a household level and access to services through referrals. Urban health extension professionals are trained at diploma-level nursing when they are recruited and are trained on the principles of an urban health extension program and assigned to households to provide door-to-door health education and related services and refer clients to health centers as necessary [27–30].

The introduction of urban health extension professionals positively changed the attitude of the majority of the households involved [31] and improved the utilization of health services in Urban Ethiopia [32]. The Urban Health Extension Program approach is based on the assumption that access and quality of primary healthcare in urban communities through the transfer of knowledge and skills to households [33]. Therefore, urban health extension workers (UHEWs) are expected to possess adequate knowledge about cervical cancer and its screening for sensitizing the community on the disease. However, little is known about the knowledge of urban health extension workers themselves towards cervical cancer screening in Ethiopia. Therefore, this study aimed to assess the level of knowledge towards cervical cancer screening and associated factors among urban health extension workers in Addis Ababa, Ethiopia.

## Methods

### Study design, setting, and population

This facility based-cross-sectional study was conducted among urban health extension workers in Addis Ababa, Ethiopia using a proportionally allocated systematic random sampling method. All urban health extension workers working in the health center at the time of data collection were included, except those who were on maternal leave. A total of 312, with a 97.5% response rate, consented and recruited from 36 health centers in Addis Ababa from February to May 2020. The sample size was calculated using a single population proportion formula based on the assumptions of the 95% confidence level, 5% margin of error, and 50% population proportion. A sample size correction formula was also used since the total number of urban health extension workers was less than 10,000. Two nurses holding a Bachelor of Science degree were involved in data collection under the supervision of two senior nurse professionals holding a Master’s of Science degree. Two days of intensive training on the content of the objective study, measuring tool and participant recruitment strategies were provided for the data collectors. To ensure the quality of the collected data the principal investigator made a continuous follow-up. The research project was reviewed and approved by an Institutional Review Board of the College of Health Sciences at Addis Ababa University. Permission to conduct the research was gained from the authorities in the study setting and written informed consents were secured from each study participant. The study was conducted following the Declaration of Helsinki.

### Measurements

The socio-demographic and reproductive characteristics of the participants were recorded using 13-items. These include residence, age, religion, educational level, work experience, marital status, age of marriage, number of children, monthly household income, age of first sexual intercourse, history of cervical cancer in the family, history of sexually transmitted infections (STIs), and history of HIV/AIDS test. The cervical cancer screening knowledge questionnaire was used to measure the knowledge of cervical cancer screening. The tool contains 14 items (5 dichotomous and 9 multiple responses), which cover the knowledge of participants regarding vulnerable groups, predisposing factors, risk factors, signs and symptoms and screening methods, treatment modality, and benefits of cervical cancer screening. Participants were asked to choose one of the two options: Yes (1) or No (1) for dichotomous questions and for multiple response *questions* we converting each item to Yes (1) or No (0). The scores ranged from 0 to 31. Participants who scored greater than or equal to the mean in the cervical cancer screening knowledge questionnaire were classified as having good knowledge about knowledge towards cervical cancer screening and those who had scored less than the mean value were considered to having poor knowledge [34, 35]. In this study, the internal consistency (*α*) of the tool used to measure knowledge of cervical cancer screening was 0.89.

Perception of cervical cancer screening was measured using 6-items. Items were scored on a 5-point Likert scale ranging from strongly disagree (1) to strongly agree (5). The score range is 6–30, with the scores greater than or equal to the mean indicating positive perception and the score less than the mean score indicating negative perception [2]. In this study, the internal consistency (*α*) of the tool to measure the perception was 0.86. The data were collected using a structured questionnaire initially developed in English and translated into Amharic version for better understanding of enumerators and the study participants. The translated Amharic version was translated back to English to ensure the meaning consistency. The instrument was pretested on study participants who were working in other health centers that were not part of the actual study. Results from the pretest were used to modify the instrument in terms of clarifying the questions.

### Data processing and analysis

The data were entered and cleaned using Epi-data version 3.5.1 and analyzed by using SPSS version 26. Frequency descriptions were computed for socio-demographic and reproductive variables and mean with standard deviation were calculated for knowledge and perception towards cervical cancer screening. The independent variables in this study were socio-demographic characteristics, reproductive characteristics, and perception of cervical cancer screening. The dependent variable was knowledge towards cervical cancer screening of the urban health extension worker. The socio-demographic variables such as age, religion, educational status, marital status, family average monthly income and work experience, reproductive variables such as the age of first sexual intercourse, parity, history of STIs, family history of cervical cancer, and history of HIV/AIDS, and perception towards cervical cancer screening association with knowledge towards cervical cancer screening were analyzed first by using a bivariate logistic regression model. Then, only those variables with *p*-values < 0.2 were taken as a candidate for multiple logistic regression analysis. In both bivariate and multiple regression models, the statistical significance of associations between variables was determined using odds ratios with 95% confidence interval and *p*-values < 0.05.

## Results

### Socio-demographic and reproductive characteristics of participants

The response rate was 97.5% with 312 out of 320 UHEWs completing the questionnaire. The mean age was 20.41 (SD = 3.73 years). The majority were Ethiopian Orthodox Christians, lived in Addis Ababa, aged between 20 and 29 years, had a diploma, and had less than 2 years of work experience. One hundred seventy-two (55.1%) participants were married at the time of data collection in which 172 (95.5%) were above the age of 18 years at their first marriage. About 47.8% of respondents never gave birth and only 11.72% started sexual intercourse before the age of 18 years. Between February to May 2020, the average monthly income of about 145 (46.5%) respondents was less than 5000ETB. Only 12 (3.8%) had a family history of cervical cancer, 8 (2.6%) had a history of STI, and 38 (12.2%) never tested for HIV (Table 1).

**Table 1:** Socio-demographic and reproductive characteristics of study participants by knowledge about CCS score (*n* = 312)

Variables	Category	N	%	Knowledge about CCS score, Mean (SD)
**Residence**	Addis Ababa	301	96.5	20.4(3.78)
	Out of Addis Ababa	11	3.5	20.5 (2.54)
**Age (**mean **=** 20.41, SD = 3.73**)**	20–29	204	64.4	20.4(3.75)
	30–39	104	33.3	20.5 (3.77)
	≥40	4	1.3	18.0 (1.41)
**Religion**	Orthodox	208	66.7	20.4(3.62)
	Protestant	69	22.1	20.5 (4.06)
	Muslim	28	9	19.6 (2.94)
	Catholic	7	2.2	20.6 (4.10)
**Level of education**	Diploma	236	75.6	20.4(3.82)
	BSc	76	24.4	20.4(3.48)
**Work experience**	1–2 years	119	38.1	19.9 (3.75)
	3–4 years	67	21.5	21.4(3.81)
	5–6 years	43	13.8	21.1 (3.52)
	> 6 years	83	26.6	19.9 (3.61)
**Marital status**	Single	130	41.7	20.6 (3.85)
	Married	172	55.1	20.3 (3.69)
	Others	10	3.2	19.9 (3.52)
**Age at first marriage (*n*** **= 182)**	< 18 years	10	5.5	18.5 (2.47)
	≥18 years	172	94.5	20.4(3.68)
**Number of children**	No child	149	47.8	20.3 (3.69)
	1–2 children	129	41.3	20.3 (3.89)
	3–4 children	34	10.9	21.1 (3.63)
**Monthly income**	< 5000 ETB	145	46.5	20.0 (3.83)
	5000–10,000ETB	123	39.4	20.6 (3.41)
	> 10,000ETB	44	14.1	21.0 (4.42)
**Age at first sexual intercourse (*n*** **= 256)**	< 18 years	30	11.72	18.8 (2.94)
	≥18 years	226	88.28	20.7 (3.79)
**History of cervical cancer in the family**	Yes	12	3.8	20.9 (3.65)
	No	297	95.2	20.4(3.74)
	Don’t know	3	1.0	22.7 (4.73)
**History of STI**	Yes	8	2.6	20.6 (4.27)
	No	304	97.4	20.4(3.73)
**HIV/AIDS test**	Yes	274	87.8	20.3 (3.78)
	No	38	12.2	20.9 (3.45)

*CCS* Cervical cancer screening, *ETB* Ethiopian Birr, *STI* Sexually transmitted infection

### Participants knowledge and perception towards cervical Cancer screening

About two-thirds of participants mentioned having multiple sexual partners as a predisposing factor and vaginal bleeding, foul-smelling vaginal discharge, and vaginal bleeding during/after sex as the sign and symptom of cervical cancer. Almost all (94.6%) participants thought that cervical cancer is a preventable disease, 302 (96.8%) believed that cervical cancer screening is necessary for early detection and prevention of cervical cancer and 295 (94.6%) thought that cervical cancer is curable if detected early. About 297 (95.2%) knew the screening procedure of cervical cancer screening, out of these 223 (71.5%) mentioned Pap smear as cervical cancer screening method, 148 (47.8%) mentioned 5 years as the recommended time for cervical cancer, and 134 (42.9%) of the participants correctly mentioned that a woman should start cervical screening at the age of 30 years (Table 2).

**Table 2:** Knowledge and perception of the study participants towards CCS (*n* = 312)

Variables	Frequency(n)	Percent (%)
**Predisposing factors to cervical cancer**		
Having multiple sexual partners	246	78.8
Early-onset sexual intercourse	222	71.2
Cigarette smoking	155	49.7
Infection by the HPV virus	184	59.0
**Sign and symptoms of cervical cancer**		
Vaginal bleeding	228	73.1
Vaginal bleeding during/after sex	213	68.3
Foul-smelling vaginal discharge	225	72.1
Pelvic or back pain	174	55.8
Post-coital bleeding	95	30.4
**Who is at risk to develop cervical cancer**		
All women	152	48.7
Married women	146	46.8
HIV positive women	174	55.8
Sexually active women	187	59.9
**Know screening procedures of cervical cancer**		
Yes	297	95.2
No	15	48
**Screening methods (*n*** **= 297)**		
Pap smear	223	71.5
VIA	97	31.1
HPV testing	179	57.4
**Frequency of cervical cancer screening**		
Every year	115	36.9
Every three year	48	15.4
Every 5 year	149	47.8
**When women screening for cervical cancer**		
When menstruation start	52	16.7
As soon as sexually active	102	32.7
At the age of 30	134	42.9
When start having children	12	3.8
After the menopause	12	3.8
**Treatment modalities for cervical cancer**		
Herbal remedies	12	3.8
Surgery	169	54.2
Radiotherapy	168	53.8
Chemotherapy	240	76.9
Cryotherapy and LEEP	83	26.6
**The benefit of cervical cancer screening**		
Early detection	232	74.4
Early treatment	210	67.3
Early diagnosis	152	48.7
Decreasing chances of an abortion	74	23.7
**Knowledge towards CCS** (mean = 20.41, SD = 3.74)		
Good Knowledge	151	48.4
Poor Knowledge	161	51.6
**Perception towards CCS** (mean = 21.02, SD = 3.27)		
Positive perception	146	46.8
Negative perception	166	53.2

*CCS* Cervical cancer screening, *HPV* Human Papilloma Virus, *LEEP* Loop Electrosurgical Excision Procedure, *SD* Standard deviation

Mean knowledge and perception scores were calculated and scores were dichotomized into two categories. In this study, about 151 (48.4%) participants found to have good knowledge by scoring above the mean knowledge score of 20.41 (SD = 3.74), and 146 (46.6%) found to have a positive attitude towards cervical cancer screening by scoring above the mean perception score of 21.02 (SD = 3.27) (Table 2).

### Factors associated with knowledge towards cervical cancer screening

The bivariate logistic regression showed that knowledge towards cervical cancer screening exhibited a statistically significant association with work experience and the monthly income of UHEWs. UHEWs who had 5–6 years of work experience (AOR = 4.32, 95% CI: 1.71, 10.94) were about 4.32 times more likely to have good knowledge towards cervical cancer screening compared to those who had 1–2 years of work experience. UHEWs with a monthly income of 5000–10,000 ETB (AOR = 3.75, 95% CI: 1.49, 9.41) and greater than 10,000 ETB (A0R = 3.08 95% CI: 1.06, 8.98) were 3.75 and 3.08 times more likely to have good knowledge towards cervical cancer screening respectively, compared to those who had a monthly income of less than 5000 ETB (Table 3).

**Table 3:** Factors associated with knowledge towards cervical cancer screening among urban health extension worker (*n* = 312)

Variables		Knowledge of CCS		Crude Odds Ratio, COR (95% CI)	Adjusted Odds Ratio, AOR (95% CI)
		Poor	Good		
**Work experience**	1–2 years	69	50	1.00	1.00
	3–4 years	25	42	2.31 (1.25, 4.28)	1.02 (0.39, 2.65)
	5–6 years	21	22	1.44 (0.71, 2.91)	4.32 (1.71, 10.94) *
	> 6 years	46	37	1.11 (0.63, 1.95)	1.34 (0.54, 3.29)
**Number of children**	No child	82	67	1.00	1.00
	1–2 children	65	64	1.21 (0.75, 1.93)	1.18 (0.43, 3.20)
	3–4 children	14	20	1.66 (0.78, 3.56)	2.37 (0.69, 8.15)
**Age of marriage**	< 18 years	10	2	1.00	1.00
	≥18 years	82	90	5.488 (1.16,25.78)	1.62 (0.08,31.54)
**Age at 1st sexual intercourse**	< 18 years	22	8	1.00	1.00
	≥18 years	106	120	3.11 (1.33,7.28)	2.78 (0.22, 34.38)
**Monthly income**	< 5000 ETB	88	57	1.00	1.00
	5000–10,000 ETB	53	70	2.04 (1.25,3.32)	3.75 (1.49, 9.41) *
	> 10,000 ETB	20	24	1.85 (0.93, 3.65)	3.08 (1.06, 8.98) *
**Perception**	Negative	91	75	1.00	1.00
	Positive	70	76	1.32 (0.84, 2.056)	1.64 (0.85, 3.17)

*p-value < 0.05, *CCS* Cervical cancer screening, *CI* Confidence interval, *ETB* Ethiopian Birr

## Discussion

This study explored the knowledge towards cervical cancer screening and associated factors among urban health extension workers in Addis Ababa, Ethiopia, and found that about 48.4% of urban health extension workers in Addis Ababa had good knowledge of cervical cancer screening. This corroborates with previous studies in Ethiopia which were conducted in Hosana, Wolaita Zone, and Addis Ababa, and other countries like Tanzania, Turkey, and Uganda that found about half of study participants had good knowledge of cervical cancer screening [10, 36–40]. However, the finding of this study was lower than the findings of other studies conducted in Swaziland (53.5%), Tanzania (63.2), Kenya (79.8%), and Qatar (92.2%) that found more than half of the study participants had good knowledge towards cervical cancer screening [37, 41–44]. This might be related to the fact that there is a difference in the educational level of the study participants, in types of health care workers involved in the study, and place and time of the study, which might be explained by the difference in the level of knowledge about cervical cancer screening. For instance, the majority of the study participants (75.6%) in this study had a diploma educational level while participants in the other studies had a degree and our study subjects were health extension workers whereas other studies surveyed nurses. Thus, urban health extension workers require more education and training to improve their knowledge about cervical cancer screening.

In this study, urban health extension workers who had 5–6 years of work experience were more likely to have good knowledge about cervical cancer screening compared to those who had 1–2 years of work experience. This corroborates with the findings of studies from other settings that found work experience has the strongest impact on women’s knowledge of cervical cancer screening [10, 17, 19, 20]. This might be related to the fact that as work experience increases exposure to health-related education or information increases. This is also justified by the finding of this study that about half of the participants (50.80%) was heard about the cervical cancer screening methods from health care provided. Therefore, participants with lower work experience need special emphasis when designing interventions aimed at improving the knowledge towards cervical cancer screening.

In this study, urban health extension workers who had a monthly income of 5000–10,000 ETB and greater had good knowledge of cervical cancer screening compared with those who had lower monthly income. This finding is similar to other studies done in other settings that found women with a lower monthly income had poor knowledge of cervical cancer screening [10, 17, 19, 20, 45]. This implies that urban health extension workers with lower monthly income may have a greater economic (financial) burden to deal with the cost of education to develop their educational level. This might also be related to those participants who earn better monthly income were more satisfied by their income to initiated in gathering information with different information systems like join in different social media (internet at home, Facebook, Youtube, and Telegrams) and using mass media (Television and Radio). Therefore, participants with lower monthly income need special emphasis when designing and implementing interventions aimed at improving the knowledge towards cervical cancer screening of this population group. This may involve the provision of economic support through allowing sponsoring them to develop their educational level since the majority of the participants in this study had diploma (level four) educational level and giving them access to cancer-related training.

In Ethiopia, cervical CCS are provided for free. To help women use this available opportunity and contribute to the reduction of morbidity and mortality; health care providers should encourage women to get the services. The results of this study are suggestive of the need to improve the level of knowledge towards cervical screening among the health extension workers themselves. Besides, the findings of this study could provide baseline information for future studies and planning intervention programs as screening is one of the strategies towards the reduction of disease burden through early detection and treatment by manipulating women’s knowledge about cervical cancer.

### Strengths and limitations

This study has a couple of strengths. One, as to the best of our knowledge it is the first study on knowledge towards cervical cancer screening among urban health extension workers in Ethiopia. Secondly, the study had a 97.5% response rate. Our study also has some limitations. Firstly, the use of a cross-sectional design does not allow inferring causality. Prospective and experimental studies are warranted. Secondly, the use of an interviewer-administered structured questionnaire for data collection. Using this method to identify knowledge towards cervical cancer screening and associated factors among urban health extension workers might involve some risk of information concealing. Thus, future research should incorporate qualitative interviews since it lets participants liberally highlight the knowledge of cervical cancer screening.

## Conclusions

This study indicated that the knowledge towards cervical cancer screening of urban health extension workers was inadequate (48.4%), although 100% of the respondents heard about cervical cancer. Urban health extension workers’ work experiences and monthly income were found to be independent predictors of the knowledge towards cervical cancer screening of respondents. Therefore, urban health extension workers with low work experiences and those with small monthly income could be targeted for cervical cancer screening information and training interventions.

## Data Availability

Datasets used and/or analyzed during the current study are available from the corresponding author on reasonable request.

## Abbreviations

AOR: Adjusted odds ratio
CC: Cervical Cancer
CCS: Cervical Cancer Screening
CI: Confidence interval
COR: Crude Odds Ratio
ETB: Ethiopian birr
HIV/AIDS: Human immunodeficiency virus/acquired immune deficiency syndrome
HPV: Human papillomavirus
LEEP: Loop Electrosurgical Excision Procedure
SD: Standard deviation
STIs: Sexually transmitted infections
UHEWs: Urban health extension workers
VIA: Visual inspection with acetic acid
WHO: World Health Organization
